# A dopamine D1-like receptor-specific agonist improves the survival of septic mice

**DOI:** 10.1016/j.isci.2024.109587

**Published:** 2024-03-27

**Authors:** Koichi Tanaka, Mohammed E. Choudhury, Satoshi Kikuchi, Ikuko Takeda, Kensuke Umakoshi, Noriyuki Miyaue, Kanta Mikami, Ayane Takenaga, Harumichi Yagi, Rintaro Shinabe, Hironori Matsumoto, Hajime Yano, Masahiro Nagai, Jun Takeba, Junya Tanaka

**Affiliations:** 1 Advanced Emergency and Critical Care Center, Ehime Prefectural Central Hospital, Kasugamachi, Matsuyama, Ehime 790-0024, Japan; 2 Department of Molecular and Cellular Physiology, Ehime University Graduate School of Medicine, Shitsukawa, Toon, Ehime 791-0295, Japan; 3 Department of Aeromedical Services for Emergency and Trauma Care, Ehime University Graduate School of Medicine, Shitsukawa, Toon, Ehime 791-0295, Japan; 4 Department of Emergency Medicine, Ehime University Graduate School of Medicine, Shitsukawa, Toon, Ehime 791-0295, Japan; 5 Department of Anatomy and Molecular Cell Biology, Nagoya University Graduate School of Medicine, Nagoya, Aichi 466-8550, Japan; 6 Division of Multicellular Circuit Dynamics, National Institute for Physiological Sciences, Okazaki, Aichi 444-8585, Japan; 7 Department of Clinical Pharmacology and Therapeutics, Ehime University Graduate School of Medicine, Shitsukawa, Toon, Ehime 791-0295, Japan

**Keywords:** Pharmacology, Natural sciences, Biological sciences, Physiology, Pathophysiology, Neuroscience, Behavioral neuroscience, Immunology

## Abstract

In this study, a murine sepsis model was developed using the cecum ligation and puncture (CLP) technique. The expression of the proinflammatory cytokines tumor necrosis factor alpha (TNF-α) and interleukin-1β (IL-1β) in the brain increased 6 h after CLP but decreased 24 h later when elevated endogenous dopamine levels in the brain were sustained. Methyl-4-phenyl-1,2,3,6-tetrahydropyridine hydrochloride reduced dopamine levels in the striatum and increased mortality in septic mice. Dopamine D1-like receptors were significantly expressed in the brain, but not in the lungs. Intraperitoneally administered SKF-81297 (SKF), a blood-brain barrier-permeable D1-like receptor agonist, prevented CLP-induced death of septic mice with ameliorated acute lung injury and cognitive dysfunction and suppressed TNF-α and IL-1β expression. The D1-like receptor antagonist SCH-23390 abolished the anti-inflammatory effects of SKF. These data suggest that D1-like receptor-mediated signals in the brain prevent CLP-induced inflammation in both the brain and the periphery.

## Highlights

• Reduced brain dopamine levels aggravated the survival of septic mice
• A D1-like receptor agonist ameliorated neuroinflammation and survival of the mice
• The agonist ameliorated lung injury, whereas the lung did not express the receptor
• Neuroinflammation may play a crucial role in sepsis-induced death

## Graphical abstract

**Figure:**
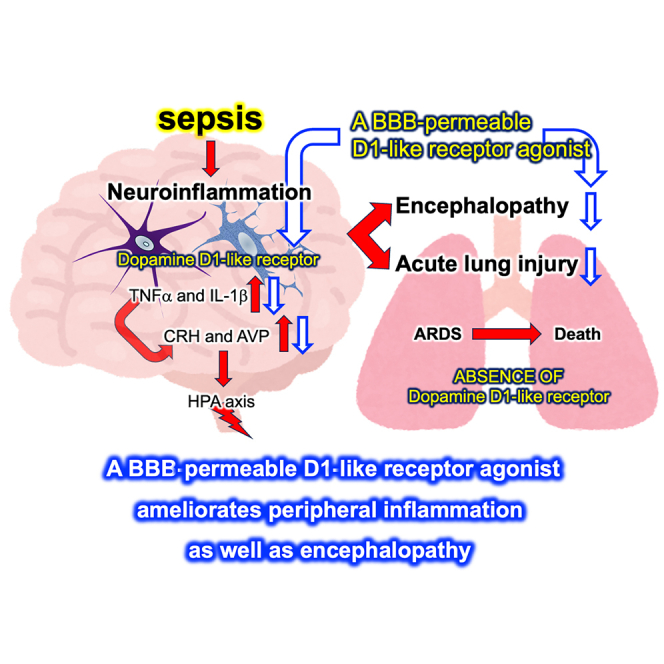

## Introduction

Sepsis is a highly fatal condition caused by an uncontrollable systemic immune reaction triggered by infection.^1^^,^^2^^,^^3^ There is no specific and highly effective treatment for sepsis, and it remains a serious cause of death in the intensive care unit (ICU). Sepsis frequently causes multiple organ failure (MOF) such as acute respiratory distress syndrome (ARDS) and acute renal failure, leading to death. In addition to dysfunction of the peripheral organs, sepsis often causes sepsis-associated encephalopathy (SAE), and it is estimated that 8%–70% of patients admitted to the ICU will develop SAE.^4^^,^^5^^,^^6^^,^^7^ Mild SAE causes only mild delirium, but severe SAE can lead to coma, which is a critical cause of increased mortality in sepsis, with mortality rates as high as 70% in cases of deep coma.^8^ Furthermore, SAE is said to cause not only acute but also long-lasting cognitive decline, leading to Alzheimer’s disease or mild cognitive impairment.^9^ Sepsis survivors often chronically suffer from depression, anxiety, post-traumatic stress disorder, and a tendency to self-harm.^10^^,^^11^

Although the pathophysiological mechanisms underlying SAE at the molecular and cellular levels have not been fully elucidated, widespread inflammation in the brain parenchyma (neuroinflammation) is considered the cause. Microglia are believed to play a critical role in inducing neuroinflammation by releasing several proinflammatory cytokines and reactive oxygen species (ROS).^12^ Sepsis-induced neuroinflammation causes breakdown of the blood-brain barrier (BBB) and subsequent infiltration of circulating leukocytes, which aggravates the pathophysiological processes of SAE.^13^ In fact, infiltrated monocytes or blood-borne brain macrophages may play a more aggravating role than resident activated microglia.^14^

Interventions to suppress the proinflammatory responses of blood-borne macrophages and activated microglia in the pathophysiological processes of SAE may be critical for ameliorating or reducing its severity.^15^^,^^16^ It is important to prevent the infiltration of circulating monocytes into the brain parenchyma. Proinflammatory cytokines, such as interleukin-1β (IL-1β) and tumor necrosis factor α (TNF-α), the major sources of which are microglia^12^ and also astrocytes,^17^ are known to cause BBB breakdown, leading to the infiltration of circulating macrophages.^18^ Elevated intracellular cyclic AMP (cAMP) levels have been shown to prevent proinflammatory activation of microglia.^19^^,^^20^ Therefore, agonists of receptors that stimulate adenylate cyclase activity are a plausible method to suppress the proinflammatory activation of microglia. Alternatively, inhibitors of phosphodiesterases that degrade cAMP may be another option.^21^ In our previous study, we showed that rat resident microglia express dopaminergic (DArgic) 1 receptor (D1R), D2R, and D4R at high levels and D3R and D5R at low levels.^22^ Dopaminergic receptors (DRs) are divided into two classes: D1-like receptors that increase intracellular cAMP levels, including D1R and D5R, and D2-like receptors that decrease cAMP levels, including D2R, D3R, and D4R.^23^^,^^24^ The selective D1-like receptor agonist SKF-81297 (SKF) elevates intracellular cAMP levels in primary cultured rat microglia and suppresses lipopolysaccharide (LPS)-induced IL-1β and TNF-α expression in cultured microglia. Even in *in vivo* experiments, SKF suppressed proinflammatory cytokine expression in the brain.

This study aimed to investigate whether SKF ameliorated SAE pathology and symptoms using a mouse sepsis model prepared by cecum ligation and puncture (CLP). Intraperitoneal administration of SKF to septic mice suppressed inflammation both in the periphery and in the brain while ameliorating SAE symptoms and mortality. Although SKF ameliorated acute lung injury (ALI)-like pathology, which may have been correlated with reduced mortality, the expression of D1-like receptors was significant in the brain, but not in the lung. Methyl-4-phenyl-1,2,3,6-tetrahydropyridine hydrochloride (MPTP) was used to decrease dopamine (DA) levels in the brain.^25^^,^^26^ MPTP-treated mice were subjected to CLP and their mortality was aggravated. The selective D1R partial agonist fenoldopam,^27^^,^^28^ which does not cross the BBB, did not show any ameliorative effect on the mortality. These results suggest that high mortality associated with sepsis may be correlated with sepsis-associated neuroinflammation.

## Results

### Septic mice viability and kinetic changes of inflammation and DA contents in the brain

**Figure 1:**
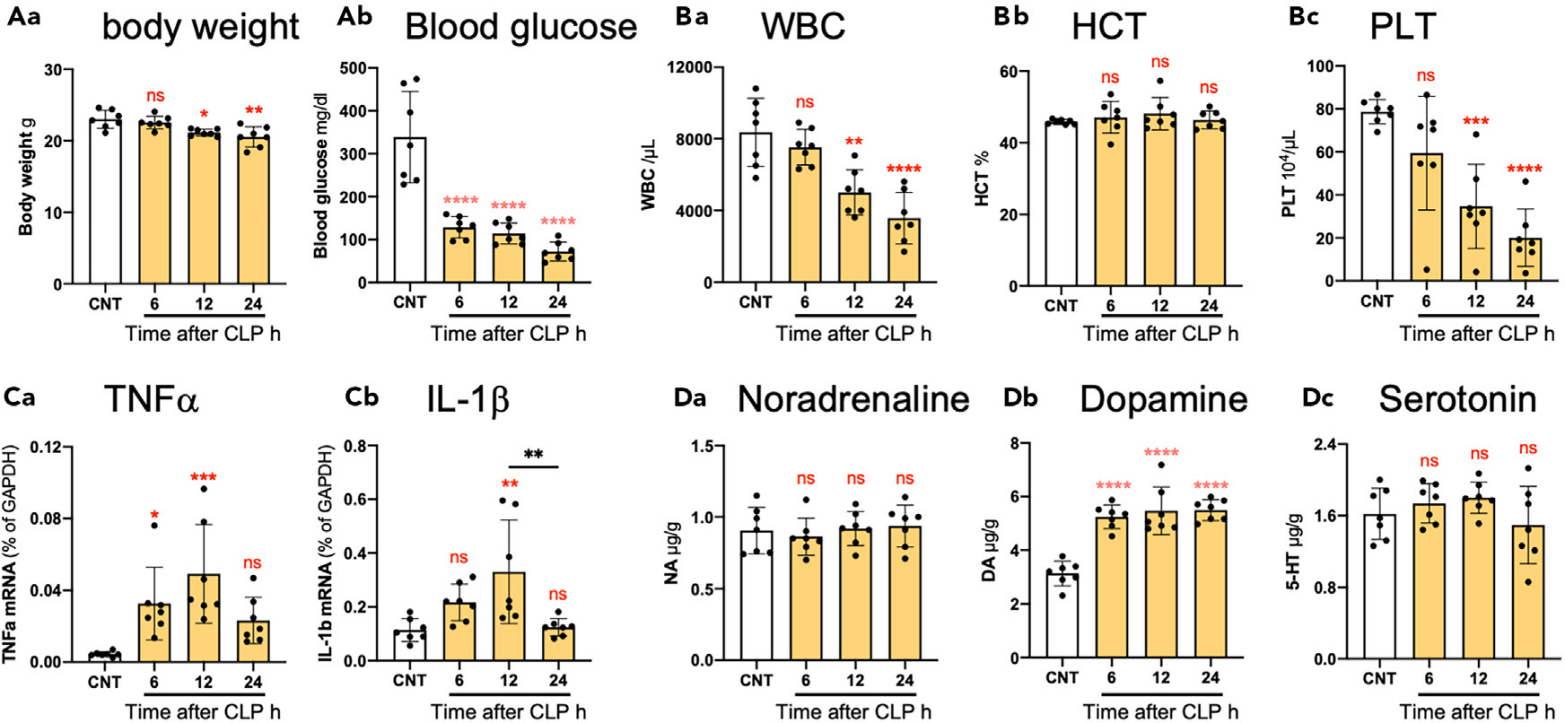
Kinetic changes in the health of the septic mice, peripheral blood cells, cytokine expression levels, and monoamine content in the brain (A) Changes in body weights of the mice (Aa) and the blood glucose levels (Ab) after CLP. CNT, healthy normal control mice. (B) Changes in the number of white blood cells (WBC; Ba), hematocrit (HCT; Bb), and the number of platelets (PLT; Bc). (C) TNF-α (Ca) and IL-1β (Cb) mRNA expression in the frontal cortex at 0, 6, 12, and 24 h after CLP. (D) Kinetic changes in noradrenaline (Da), dopamine (Db), and serotonin (Dc) contents in the frontal cortex. *n* = 7. A one-way ANOVA and Tukey’s multiple comparison test. ∗, ∗∗, ∗∗∗, ∗∗∗∗ indicate statistical significance vs. CNT at *p* < 0.05, 0.01, 0.001, and 0.0001, respectively. Asterisks and “ns” in red indicate statistical difference against CNT data. All numerical data and the relevant statistical main factors are shown in Table S1. Data are expressed as the mean ± SD.

CLP weakened mice, causing weight loss and decreased blood glucose levels (Figure 1A). The numbers of white blood cells and platelets also decreased (Figure 1B). The latter was suggestive of disseminated intravascular coagulation, a symptom of sepsis.^29^ The expression of mRNA encoding TNF-α and IL-1β in the left frontal cortex increased from 6 to 12 h after CLP, and the elevation subsided 24 h after CLP (Figure 1C), suggesting the presence of endogenous mechanisms that suppress CLP-induced neuroinflammation. The DA level in the right frontal cortex was elevated 6 h after CLP (Figure 1Db). Our previous study showed a probable correlation between elevated endogenous DA levels and suppressed TNF-α expression in an LPS-induced delirium rat model.^22^ The levels of noradrenaline (NA) and serotonin did not change significantly.

**Figure 2:**
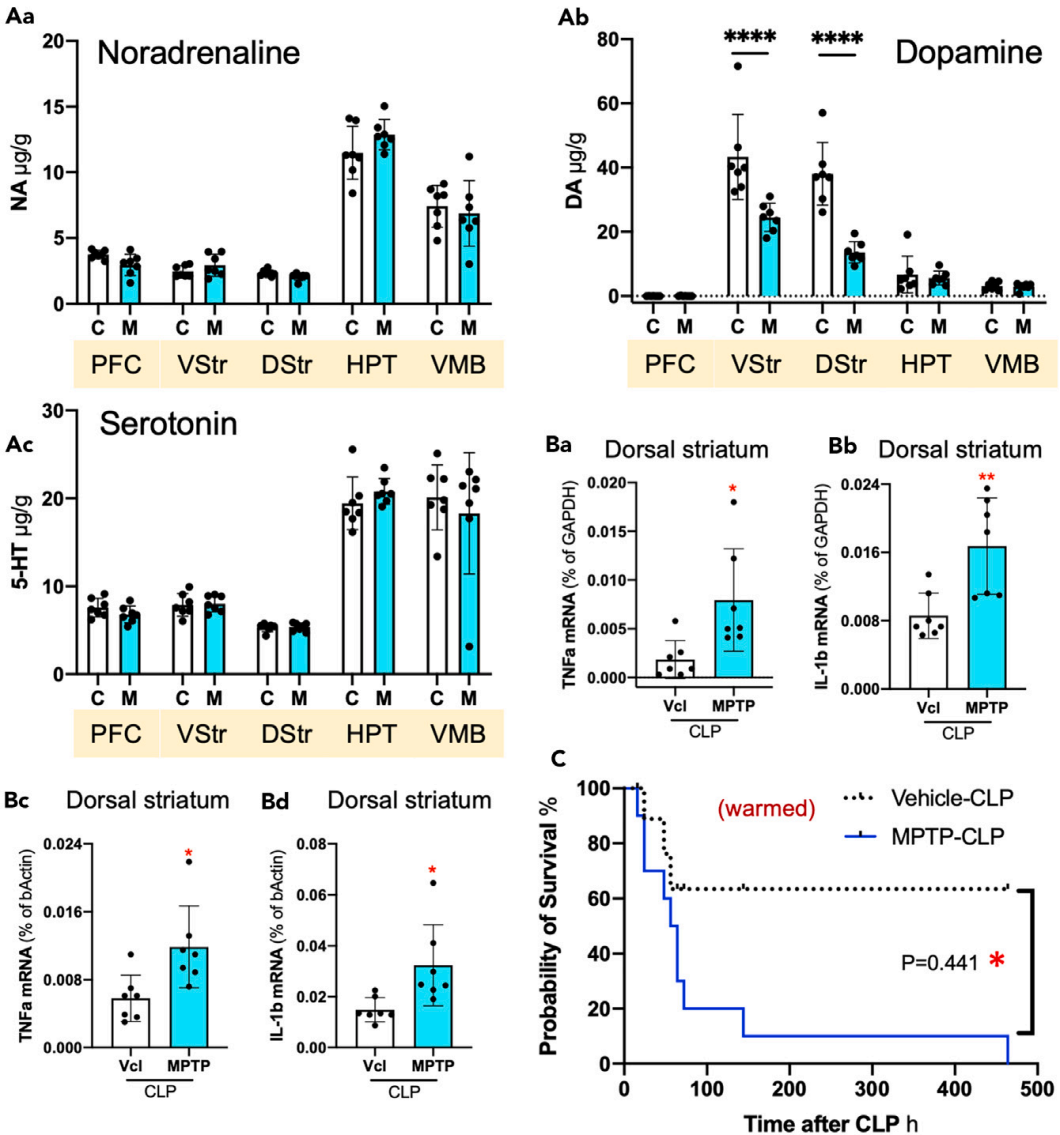
Effects of MPTP treatment on monoamine content in the brain and survival of septic mice (A) The noradrenaline (Aa), dopamine (Ab), and serotonin (Ac) content in the PFC, VStr, DStr, HPT, and VMB of normal control mice shown as “C” in the graph and the MPTP-treated mice as “M”. (B) TNF-α (Ba, Bc) and IL-1β (Bb, Bd) mRNA expression in the DStr of CNT and MPTP-treated mice. Data of Ba and Bb are normalized to the GAPDH mRNA expression, while data of Bc and Bd are normalized to the β-actin mRNA expression. *n* = 7. A one-way ANOVA and Tukey’s multiple comparison test. ∗, ∗∗, ∗∗∗∗ indicate statistical significance at *p* < 0.05, 0.01, and 0.0001, respectively. (C) Kinetic changes in the survival rate after CLP in comparison to vehicle- and MPTP-treated mice. The mice were warmed for 1 h after CLP to prevent hypothermia. *n* = 10. Data are expressed as the mean ± SD. Log rank test (Mantel-Cox) test. ∗∗ indicates statistical significance at *p* < 0.01. Asterisks and “ns” in red indicate statistical difference against CNT data. All numerical data and the relevant statistical main factors are shown in Table S1.

MPTP is a chemical frequently used to prepare Parkinson’s disease mouse models because of its toxicity to DA neurons.^25^^,^^26^ After the intraperitoneal administration of MPTP, the prefrontal cortex (PFC), ventral striatum (VStr) containing the nucleus accumbens, dorsal striatum (DStr), hypothalamus (HPT), and ventral midbrain (VMB) containing the substantia nigra and ventral tegmental area were dissected and subjected to monoamine determination by high-performance liquid chromatography (Figure 2A). MPTP decreased DA levels in the VStr and DStr but not in the other brain regions. The TNF-α and IL-1β mRNA expression in the DStr in MPTP-treated/CLP-induced septic (MPTP/CLP) mice was increased in comparison to vehicle-treated/CLP-induced septic (Vcl/CLP) mice (Figure 2B). All MPTP/CLP-treated mice died after CLP within 20 days, while 63.5% of control CLP-treated mice survived, suggesting that endogenous DA plays a role in the prevention of death (Figure 2C). In addition, the mice shown in Figure 2C were warmed to prevent CLP-induced hypothermia, which ameliorated the survival of septic mice.^30^

### Expression of DA receptors in the brain and lung

**Figure 3:**
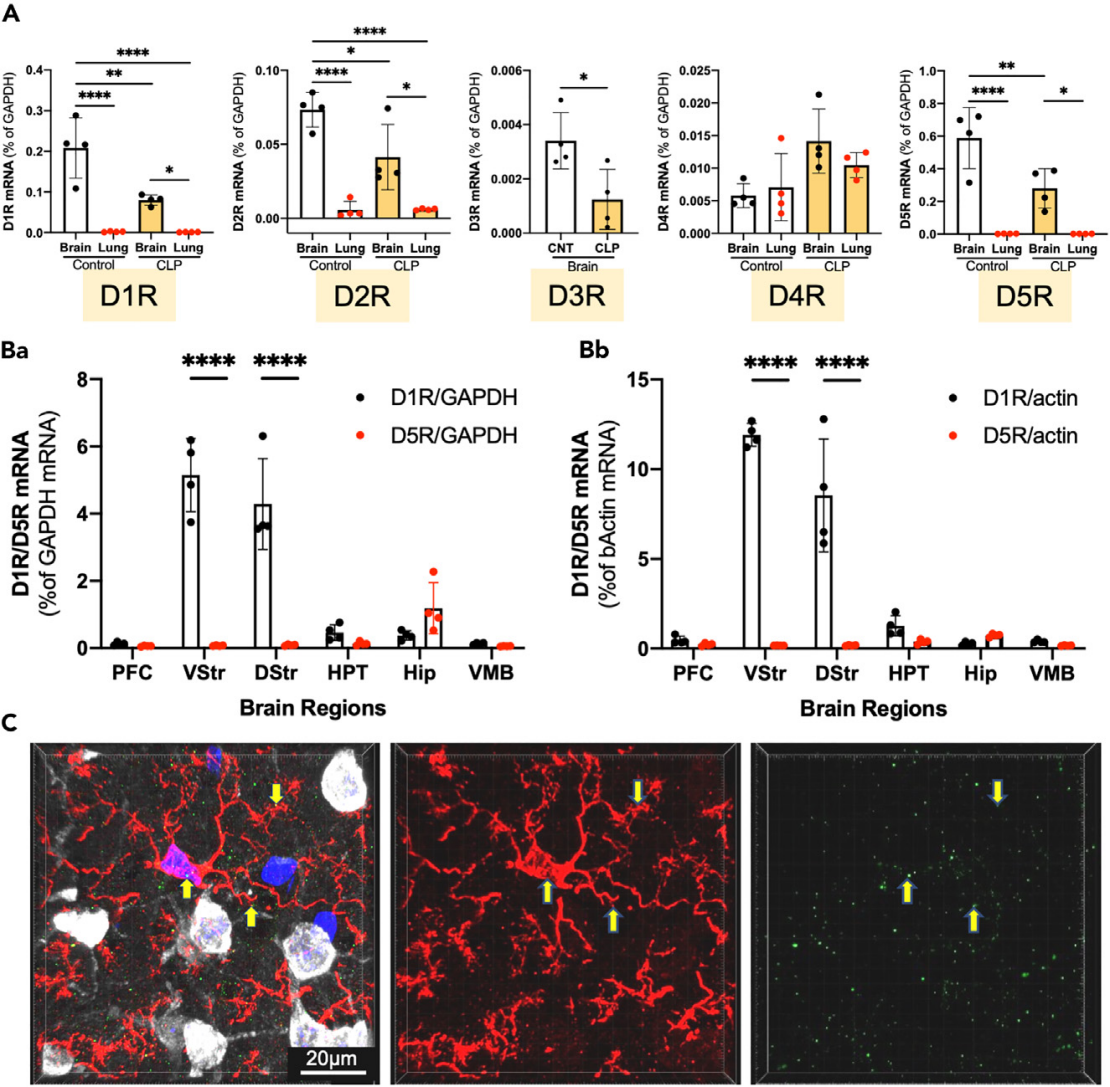
The expression of dopamine receptors (A) Total RNA was obtained from the normal control mouse brain and lung as well as the CLP-induced septic mouse brain and lung. cDNA was prepared from the total RNA and subjected to qPCR to determine mRNA levels for DRs. mRNA for D3R in the lung was not detected. *n* = 4. A one-way ANOVA and Tukey’s multiple comparison test. (B) Distribution of mRNA for D1R and D5R in the PFC, VStr, DStr, HPT, Hip, and VMB of normal control mice as revealed by qPCR. Data of Ba are normalized to the GAPDH mRNA expression, while data of Bb are normalized to the β-actin mRNA expression. *n* = 4. The expression of D1R was significantly higher than that of D5R in the VStr and DStr. Data are expressed as the mean ± SD. A two-way ANOVA and Sidak’s multiple comparison test. ∗, ∗∗, ∗∗∗∗ indicate statistical significance at *p* < 0.05, 0.01, and 0.0001, respectively. (C) Immunohistochemical staining revealed that D1R protein was localized in the microglia in the frontal cortex of normal control mice. Green, D1R; red, CD11b; red, white; NeuN, blue; Hoechst33324. Arrows denote green fluorescence localized on a microglial cell. See Video S1. All numerical data and the relevant statistical main factors are shown in Table S1.

DR expression 6 h after CLP was investigated using cDNA prepared from the brain (left frontal cortex) and lung tissues of the normal control and CLP-treated septic mice (Figure 3A). D1R mRNA was more strongly expressed in the brain than in the lungs. Similar results were obtained for both D2R and D5R. No D3R expression was detected in the lungs. CLP reduces D1R, D2R, D3R, and D5R expression in the brain. The expression of D1R and D5R mRNA was analyzed using cDNA prepared from dissected brain regions of normal control mice, which included PFC, VStr, DStr, HPT, hippocampus (Hip), and VMB (Figure 3B). D1R mRNA was expressed in the striatum (VStr and DStr) at much higher levels than in the other brain regions. The expression of D5R mRNA was much lower than that of D1R mRNA in the striatum. The D1R protein expression in the microglia in the PFC was confirmed by an immunohistochemical analysis (Figure 3C; Video S1).

Video S1. Expression of D1R by a microglial cell in the frontal cortex, related to Figure 3

### SKF prevented death of septic mice while suppressing inflammatory changes in circulation

**Figure 4:**
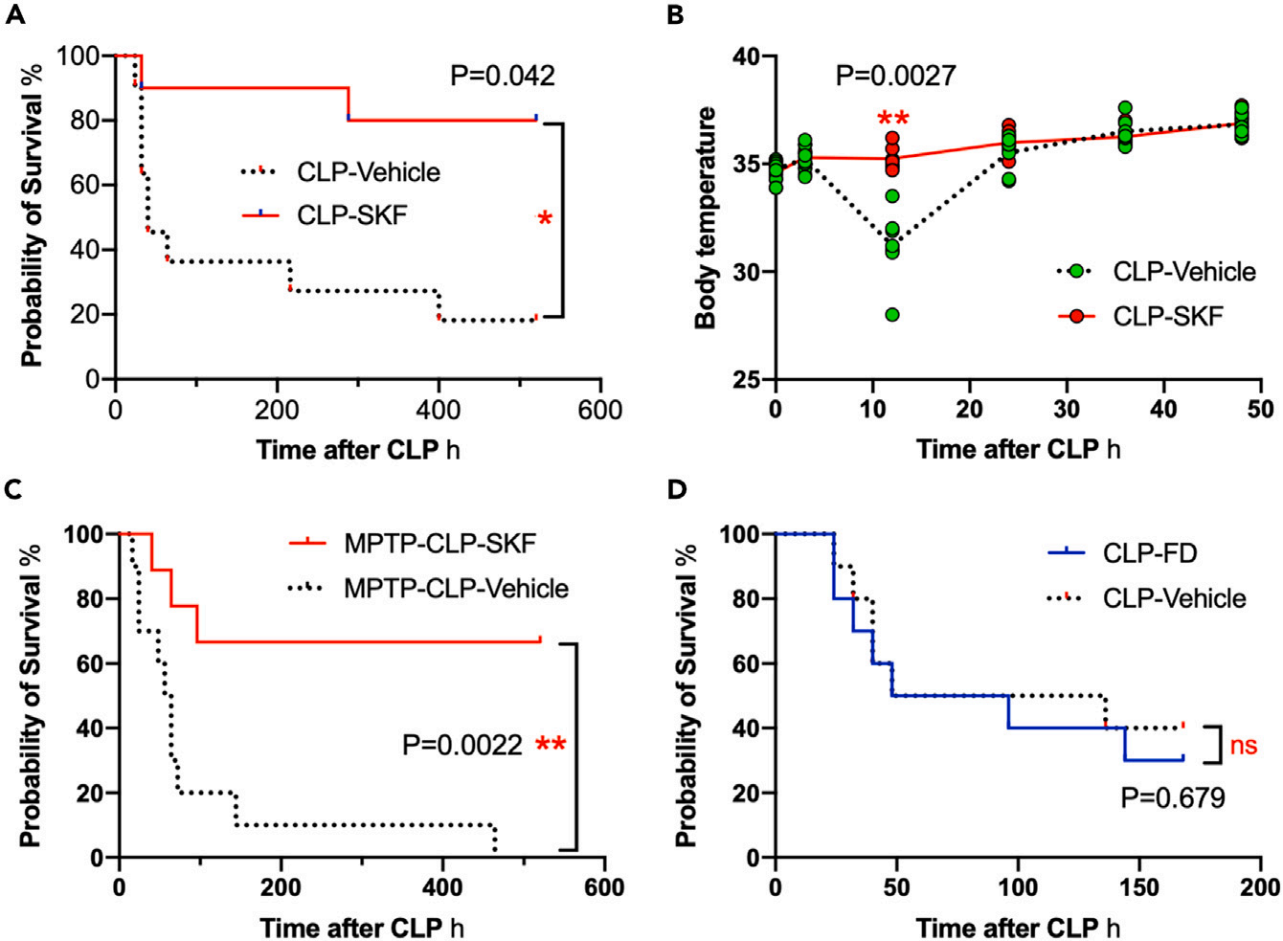
Effects of SKF and fenoldopam on the septic mouse survival (A) SKF prevented death of CLP-induced septic mice. CLP-Vehicle group *n* = 11; CLP-SKF group *n* = 10. Log rank test (Mantel-Cox) test. (B) Changes in body temperature of surviving septic mice from 0 to 48 h after CLP. *n* = 7. A two-way ANOVA with Sidak’s multiple comparison test. (C) SKF prevented the death of MPTP-treated septic mice. Vehicle-treated group *n* = 10; SKF-treated group *n* = 9. Log rank test (Mantel-Cox) test. (D) Fenoldopam (FD) did not change the survival rate after CLP. Each group *n* = 10. Log rank test (Mantel-Cox) test. ∗, ∗∗ indicate statistical significance at *p* < 0.05, 0.01, respectively. Asterisks in red indicate the significant differences against CNT data. All numerical data and the relevant statistical main factors are shown in Table S1.

This study employed SKF as a BBB-permeable D1-like receptor agonist, which has been reported to prevent LPS-induced proinflammatory activation of rat microglia *in vitro* and *in vivo.*^22^ First, we investigated whether SKF ameliorated CLP-induced sepsis by investigating changes in overall survival and body temperature. The mortality rate of CLP-induced septic mice reached 80%. When SKF (1 mg/kg body weight) was intraperitoneally administered once per day for 7 consecutive days, starting shortly after CLP, the mortality rate was reduced to 13% (Figure 4A). Marked transient hypothermia was observed 12 h after CLP in Vcl/CLP mice, whereas hypothermia was prevented entirely in SKF-treated/CLP-treated (SKF/CLP) mice (Figure 4B). To distinguish between the central and peripheral effects of DR agonists, the effects of the D1R agonist fenoldopam, which does not cross the BBB,^28^^,^^31^ were compared with those of SKF. SKF ameliorated the survival of MPTP-treated septic mice (Figure 4C), suggesting that SKF compensated for the loss of endogenous DA effects in the brain. Fenoldopam (10 mg/kg body weight) did not affect the survival of septic mice (Figure 4D).

### SKF suppressed CLP-induced peripheral inflammation

**Figure 5:**
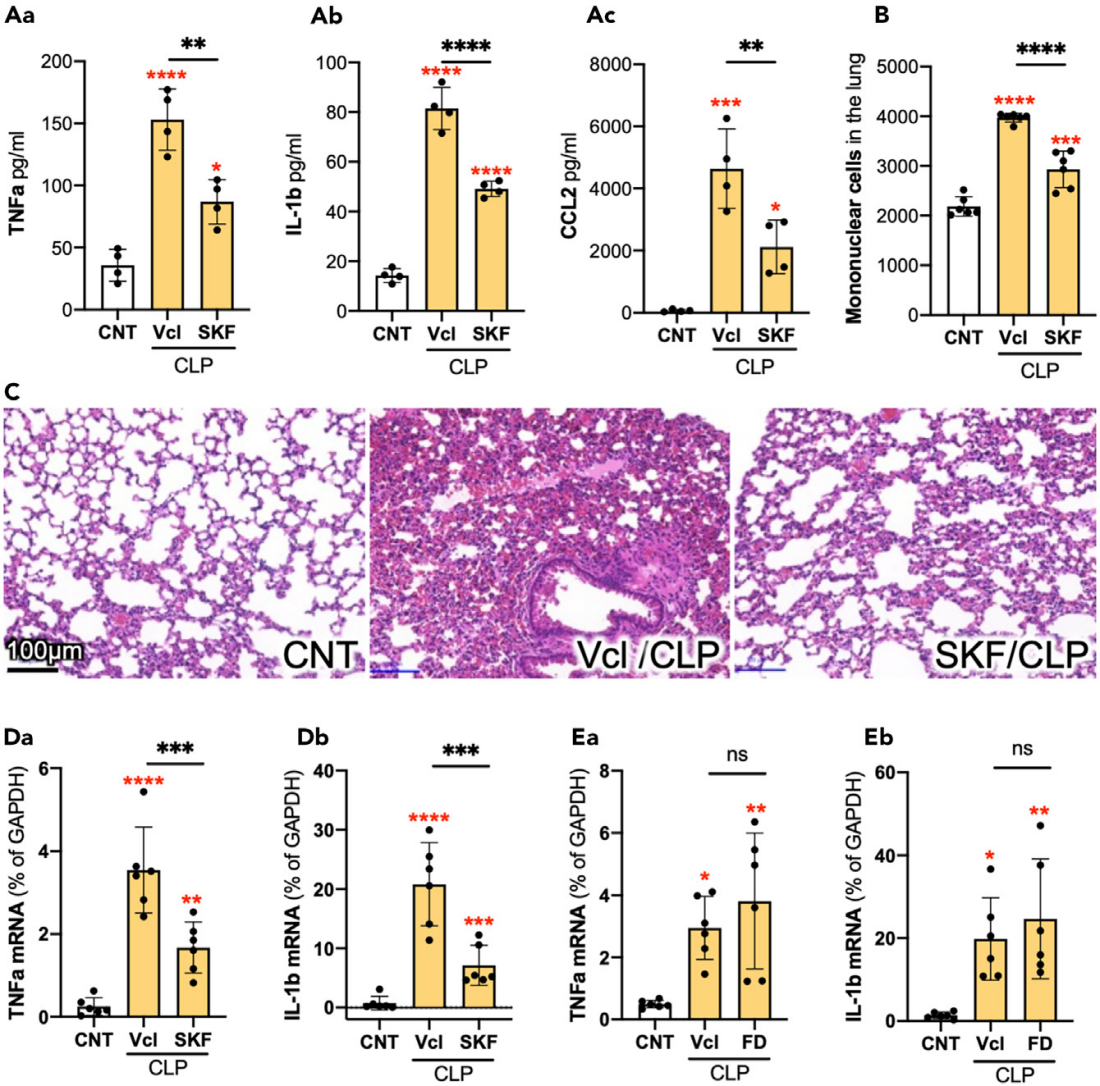
SKF suppressed systemic inflammation (A) Determination by an ELISA of the CCL2 (Aa), TNF-α (Ab), and IL-1β (Ac) levels in the peripheral blood collected from normal healthy control mice (CNT), vehicle administered CLP-treated mice (Vcl/CLP), and SKF administered CLP-treated mice (SKF/CLP) 6 h after CLP. *N* = 4. A one-way ANOVA with Tukey’s multiple comparison test. (B) The number of mononuclear cells infiltrated into the lungs 6 h after CLP. *n* = 6. A one-way ANOVA with Tukey’s multiple comparison test. (C) Representative H&E-stained lungs of CNT, Vcl/CLP, and SKF/CLP. Micrographs of all H&E-stained specimens (*n* = 6) are shown in Figure S1. (D) mRNA expression encoding TNF-α (Da) and IL-1β (Db) in the lung of CNT, Vcl/CLP, and SKF/CLP mice 6 h after CLP. *n* = 7. A one-way ANOVA with Tukey’s multiple comparison test. (E) mRNA expression encoding TNF-α (Ea) and IL-1β (Eb) in the lung of CNT, Vcl/CLP, and FD/CLP mice 6 h after CLP. *N* = 6. Data are expressed as the mean ± SD. A one-way ANOVA with Tukey’s post hoc test. ∗, ∗∗, ∗∗∗, ∗∗∗∗ indicate statistical significance at *p* < 0.05, 0.01, 0.001, and 0.0001, respectively. Asterisks in red indicate the significant differences against CNT data. All numerical data and the relevant statistical main factors are shown in Table S1.

Concentrations of TNF-α, IL-1β, and CCL2 in peripheral blood collected 6 h after CLP were determined by ELISA (Figure 5A). CLP increased the levels of cytokines and chemokines, whereas the administration of SKF decreased their levels. Sepsis causes ALI, which often leads to ARDS, a critical cause of sepsis-associated death.^32^^,^^33^ Flow cytometry analyses revealed that CLP enhanced the infiltration of mononuclear cells into the lung tissues and that SKF prevented this infiltration (Figure 5B). As shown by H&E staining, the sepsis model also displayed ALI-like pathological changes (Figures 5C and S1). In the lungs of Vcl-treated septic (Vcl/CLP) mice, the alveolar walls were thickened, and a large number of infiltrated mononuclear cells were observed in the tissues. SKF prevented thickening of the alveolar walls (Figure 5C; SKF/CLP). The expression of TNF-α and IL-1β mRNA increased in the lung tissues of Vcl/CLP mice, and this increase was prevented by the administration of SKF (Figure 5D). In contrast, fenoldopam did not suppress the CLP-induced increase in the expression of cytokines in lungs (Figure 5E).

### SKF suppressed neuroinflammation and an antagonist abolished its effects

**Figure 6:**
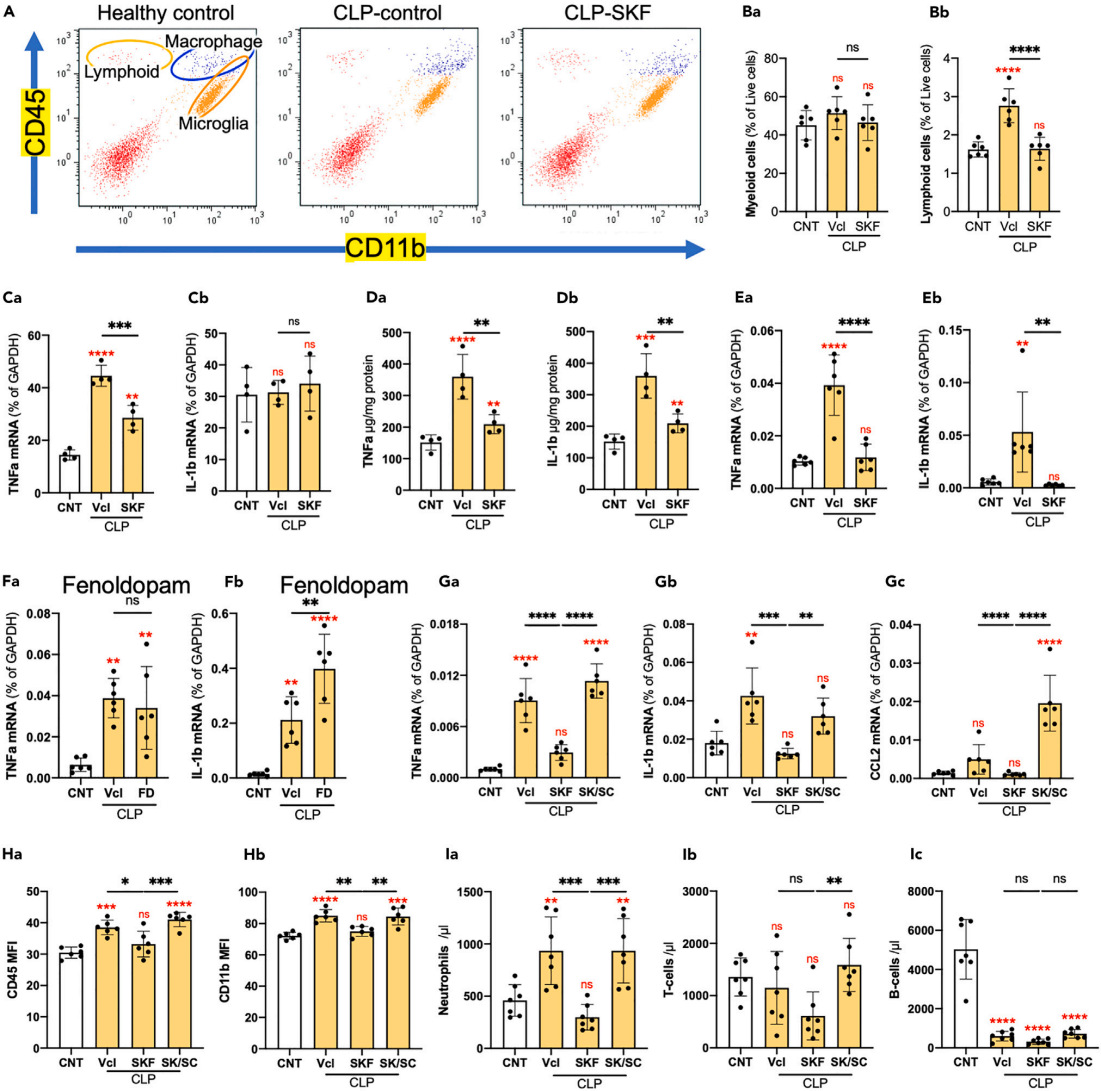
Effects of SKF and D1-like receptor antagonist SCH on CLP-induced neuroinflammation and circulating leukocytes All data shown here were obtained using samples obtained 12 h after CLP. (A) Representative flow cytometry plots of dissociated frontal cortex cells. Using anti-CD11b and anti-CD45 antibodies, lymphoid cells, blood-borne macrophages, and resident microglia were separated. (B) Flow cytometry analyses of the percentage in the total live cells of CD11b^+^/CD45^+^ myeloid cells (Ba) and CD11b^−^/CD45^+^ lymphoid cells (Bb). *n* = 6 or 7. (C) Effects of CLP and SKF on the mRNA expression of TNF-α (Ca), IL-1β (Cb) by sorted microglia. *n* = 4. (D) Effects of CLP and SKF on the protein expression of TNF-α (Da) and IL-1β (Db) in the hippocampus. (E) Effects of CLP and SKF on the mRNA expression of TNF-α (Ea) and IL-1β (Eb) in the hippocampus. *n* = 6. (F) Effects of FD on TNF-α (Fa) and IL-1β (Fb) mRNA expression in the frontal cortex. *n* = 6. (G) Effects of SKF and SKF+SCH (SK/SC) on the mRNA expression of TNF-α (Ga) and IL-1β (Gb), and CCL2 (Gc) in the frontal cortex of CLP-treated mice. See also Figure 2S. *n* = 6. (H) Effects of SKF and SK/SC on the CD45 (Ha) and CD11b (Hb) expression by microglia/macrophages as revealed by FACS. *n* = 6. (I) Effects of SKF and SK/SC on circulating neutrophils (Ia), T cells (Ib), and B cells (Ic). *n* = 7. Data are expressed as the mean ± SD. A one-way ANOVA with Tukey’s multiple comparison test. ∗, ∗∗, ∗∗∗, ∗∗∗∗ indicate statistical significance at *p* < 0.05, 0.01, 0.001, and 0.0001, respectively. Asterisks and “ns” in red indicate statistical difference against CNT data. All numerical data and the relevant statistical main factors are shown in Table S1.

We investigated whether SKF suppressed CLP-induced neuroinflammation using flow cytometry 12 h after CLP (Figure 6). Neither CLP treatment nor the administration of SKF significantly affected the percentage of CD11b^+^/CD45^+^ myeloid cells in the brain, the majority of which were resident microglia and blood-borne macrophages (Figure 6Ba); however, the percentage of CD11b^−^/CD45^+^ lymphoid cells increased in Vcl/CLP mice but not in SKF/CLP mice (Figure 6Bb). Increased TNF-α but not IL-1β mRNA expression in microglia sorted from the entire forebrain was observed, and SKF reduced TNF-α expression (Figure 6C). CLP also elevated TNF-α and IL-1β expression in the hippocampus at both protein (Figure 6D) and mRNA (Figure 6E) levels. SKF overcame these proinflammatory changes in the hippocampus. The BBB-impermeable D1-receptor agonist fenoldopam did not affect CLP-induced changes in TNF-α but increased the IL-1β mRNA expression in the frontal cortex (Figure 6F). Furthermore, we investigated whether SCH-23390 hydrochloride (SCH; 1 mg/kg body weight), a D1-like dopamine receptor antagonist,^27^ abolished the effects of SKF. The TNF-α and IL-1β mRNA expression increased in the frontal cortex 12 h after CLP, and SKF suppressed the proinflammatory changes (Figures 6G and S2). The simultaneous administration of SCH and SKF abolished the anti-inflammatory effects of SKF. CLP did not markedly increase the expression of CCL2 mRNA, whereas the simultaneous administration of SKF and SCH increased the expression of CCL2 mRNA. SKF almost abolished the CLP-induced elevation in the mean fluorescence intensity (MFI) of CD45 and CD11b in myeloid cells (Figure 6H). SCH abolished the effects of SKF on MFI changes. The peripheral effects of SCH on SKF were also investigated. CLP increased the number of neutrophils in the circulation, SKF overcame these changes, and SCH abolished the SKF effect (Figure 6I). In contrast, CLP did not significantly affect the number of circulating T cells. As described elsewhere,^30^ CLP markedly reduced the number of B cells in the circulation; SKF and SCH did not cause any apparent changes.

### Effects of SKF on cognitive dysfunction and diurnal rhythms of septic mice

**Figure 7:**
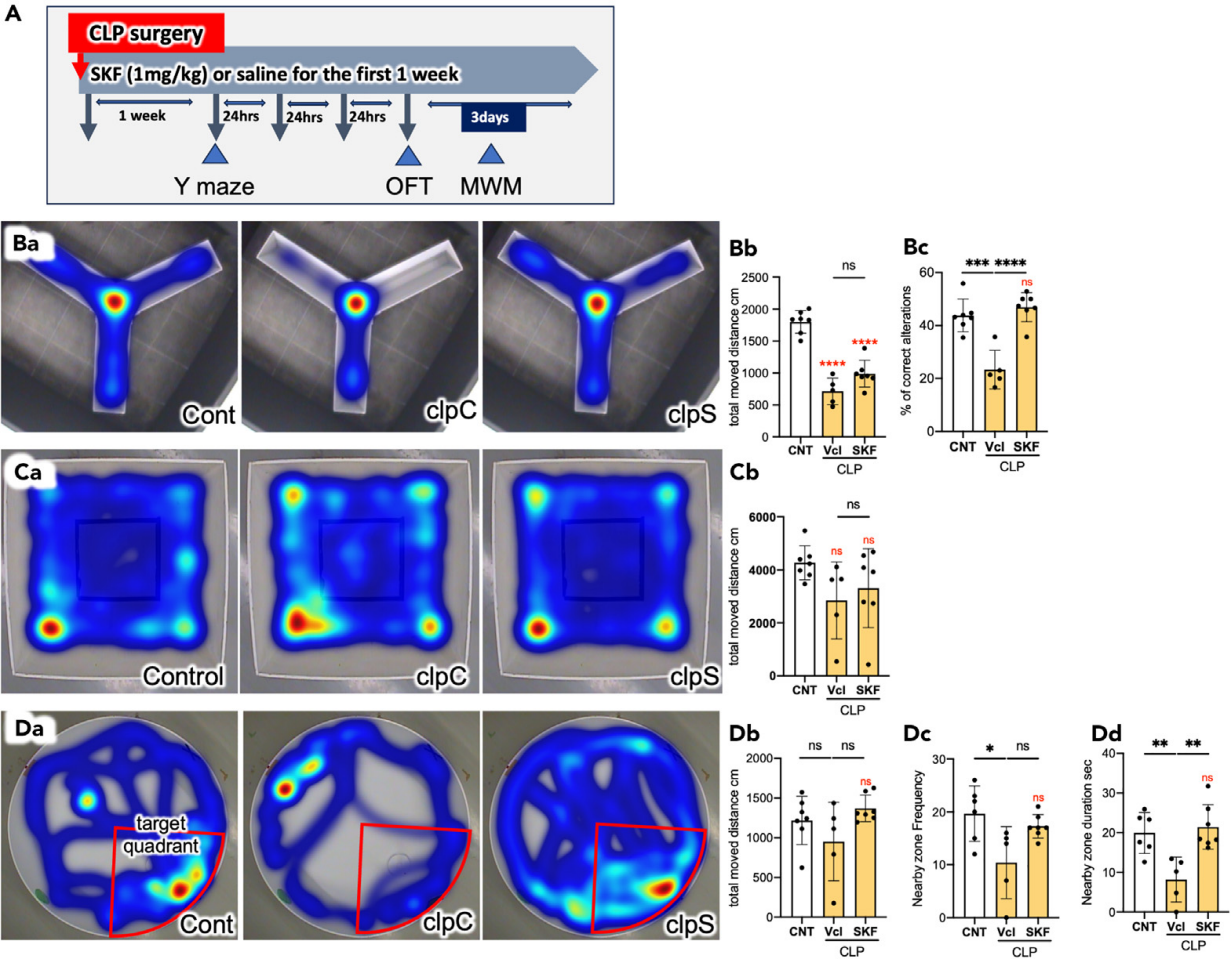
SKF ameliorates CLP-induced cognitive dysfunction (A) Experimental protocol for behavioral tests. Shortly after CLP, administration of SKF or vehicle was started once per day for a week, and the Y-maze test was conducted a week after CLP. Ten days later, the open-field test (OFT) was performed, followed by the Morris water maze (MWM) test for 3 days. (B) (Ba) Representative heatmaps of the Y-maze test. (Bb) Total moved distance and (Bc) percentage of correct alternative movements. *n* = 7. (C) Mobile activities evaluated by the OFT. (Ca) Representative heat maps for the OFT results. (Cb) Mobile activities of clpC and clpS mice did not significantly differ from normal control mice (CNT). CNT and SKF/CLP; *n* = 7, Vcl/CLP; *N* = 5. (D) Results of the MWM test. (Da) Representative heat maps, (Db) total moved distance, (Dc) frequency entering the nearby zone, and (Dd) duration in the nearby zone. *n* = 6 (CNT), 5 (Vcl/CLP), and 7 (SKF/CLP). Data are expressed as the mean ± SD. A one-way ANOVA with Tukey’s multiple comparison test. ∗, ∗∗, ∗∗∗, ∗∗∗∗ indicate statistical significance at *p* < 0.05, 0.01, 0.001, and 0.0001, respectively. Asterisks and “ns” in red indicate statistical difference against CNT data. All numerical data and the relevant statistical main factors are shown in Table S1.

Sepsis frequently causes cognitive dysfunction, a typical symptom of SAE.^6^^,^^7^^,^^13^ The cognitive function of the mice was evaluated using Y-maze (Figure 7B) and Morris water maze (MWM) (Figure 7D) tests. In the Y-maze test, both Vcl/CLP and SKF/CLP mice displayed a decreased total distance moved (Figure 7Bb), which might be related to disturbed cognitive functions rather than motor function deficits, because the open-field test showed no significant differences in the total distance moved between the normal control (CNT), Vcl/CLP, and SKF/CLP groups (Figure 7C). Furthermore, the percentage of correct alterations in the Y-maze test decreased in Vcl/CLP mice, which is indicative of cognitive dysfunction (Figure 7Bc). Administration of SKF restored the correct alteration ratio to the control level. The total distance moved during the MWM test did not differ significantly among the three groups (Figure 7Db). The MWM test revealed that Vcl/CLP mice displayed a decreased frequency of crossing the border of the nearby zone around the position where the platform had been placed the previous day (Figure 7Dc). Vcl/CLP mice also showed a decreased duration in the nearby zone (Figure 7Dd).

**Figure 8:**
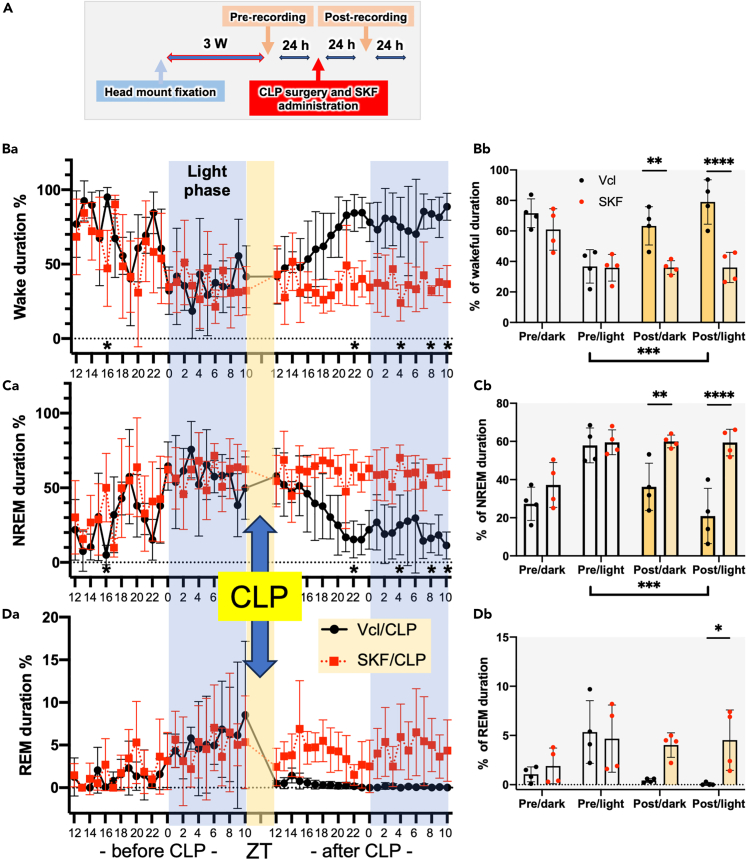
EEG/EMG recordings showing changes diurnal rhythms after CLP (A) Experimental protocol for EEG/EMG recordings. The duration percentages for wakefulness (B), NREM (C), and REM (D). Hourly data (a) and the total duration of the dark and light phases (b) are shown. n = 4. Data are expressed as the mean ± SD. A two-way ANOVA with Sidak’s multiple comparison test. ∗, ∗∗, ∗∗∗, ∗∗∗∗ indicate statistical significance at *p* < 0.05, 0.01, 0.001, and 0.0001, respectively. All numerical data and the relevant statistical main factors are shown in Table S1.

CLP disturbed the diurnal rhythm of the mice, as revealed by the electroencephalogram (EEG)/electromyogram (EMG) recordings (Figure 8). Before CLP, Vcl/CLP and SKF/CLP mice displayed similar diurnal rhythms, with prolonged wakefulness and sleep durations during the dark and light phases, respectively. After CLP, the Vcl/CLP mice displayed an increased duration of wakefulness or insomnia during the light period. In contrast, SKF/CLP mice showed prolonged sleep duration and disturbed diurnal rhythm. Rapid eye movement (REM) and non-REM (NREM) sleep were distinguished using EEG/EMG. SKF appeared to prolong both REM and NREM durations.

### CLP-induced changes in HPA axis

**Figure 9:**
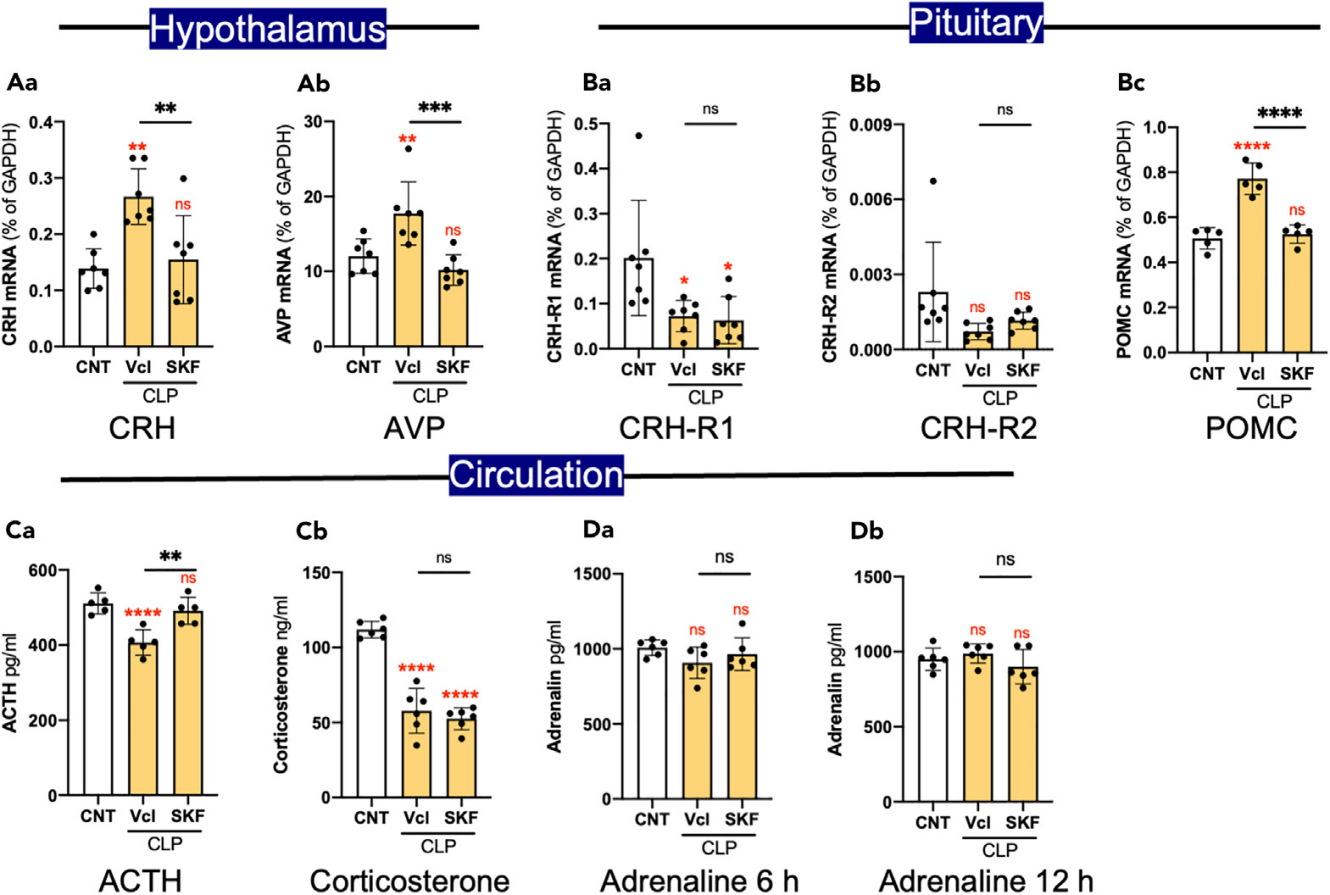
Effects of CLP and SKF on the HPA axis and hormone levels (A) Changes in expression of mRNA encoding CRH (Aa) and AVP (Ab) in the HPT 12 h after CLP. *n* = 7. (B) CRH-R1 (Ba), CRH-R2 (Bb), and POMC (Bc) mRNA expression in the pituitary gland 12 h after CLP. *n* = 6. (C) ACTH (Ca; *n* = 5) and corticosterone (Cb; *n* = 6) levels in the circulation as revealed by ELISA 12 h after CLP. (D) Adrenaline levels in the circulation 6 (Da) and 12 h (Db) after CLP as revealed by ELISA. Data are expressed as the mean ± SD. A one-way ANOVA with Tukey’s multiple comparison test. ∗, ∗∗, ∗∗∗∗ indicate statistical significance at *p* < 0.05, 0.01, and 0.0001, respectively. Asterisks and “ns” in red indicate statistical difference against CNT data. All numerical data and the relevant statistical main factors are shown in Table S1.

Sepsis has been reported to cause disturbances in the hypothalamic-pituitary-adrenal axis (HPA axis).^34^ The HPT and pituitary were dissected from CNT, Vcl/CLP, and SKF/CLP mice 12 h after CLP. The expression of corticotropin-releasing hormone (CRH) and arginine vasopressin (AVP) mRNA was increased in the HPT of Vcl/CLP mice, and this increase was abolished by the administration of SKF (Figure 9A). The expression of CRH-receptor 1 (CRH-R1) in the pituitary of Vcl/CLP and SKF/CLP mice was decreased in comparison to CNT mice, while no significant changes were observed in the pituitary expression of CRH-R2 mRNA (Figure 9B). The mRNA expression of proopiomelanocortin (POMC), a precursor of adrenocorticotropic hormone (ACTH), was increased in the pituitaries of Vcl/CLP mice, and this increase was abolished by the administration of SKF (Figure 9Bc). ACTH levels in circulation were decreased in Vcl/CLP mice, and an ELISA demonstrated that the administration of SKF overcame this decrease (Figure 9Ca). However, corticosterone levels were similar between the Vcl/CLP and SKF/CLP mice (Figure 9Cb).^35^ Although adrenaline strongly affected CLP-induced immune reactions,^36^ no significant changes in circulating adrenaline levels were observed in CNT, Vcl/CLP, or SKF/CLP mice (Figure 9D).

## Discussion

The present study showed that the BBB-permeable D1-like receptor agonist SKF suppressed CLP-induced neuroinflammation and systemic inflammation, while markedly reducing animal death. Sepsis-induced death has mainly been attributed to MOF.^37^ Among cases of sepsis-induced MOF, ALI as a cause of ARDS may be critically involved in sepsis-induced death.^33^^,^^37^ SKF ameliorated ALI-like pathology, as revealed by the reduced accumulation of mononuclear cells, suppressed thickening of alveolar walls, and reduced TNF-α and IL-1β expression in the lung. However, it is unlikely that SKF directly suppressed inflammation and subsequently prevented the incidence of ALI, because the expression of D1R and D5R was very low in the lung. In contrast, DRs, in particular D1R rather than D5R, were abundantly expressed in the brain, suggesting that SKF primarily acts in the brain. The D1-like receptor antagonist SCH almost completely abolished the anti-inflammatory effects of SKF, suggesting that the effects of SKF are mediated through D1-like receptors, but not the off-target effects of SKF. Neuroinflammation may be a critical cause of SAE, a symptom of MOF. The mortality of sepsis patients with severe SAE symptoms is markedly elevated.^6^^,^^7^^,^^13^ Taken together, the ameliorating effects of SKF on septic mouse mortality may be attributed to the amelioration of neuroinflammation.

In our previous study, endogenous DA levels increased after intraperitoneal LPS administration in rats, and the elevated DA levels correlated with the suppressed expression of TNF-α.^22^ To determine whether endogenous murine brain DA effectively suppresses CLP-induced systemic and intracerebral inflammation, MPTP treatment was used to reduce DA levels.^25^^,^^26^ MPTP-treated mice died more frequently in response to CLP than control septic mice. MPTP specifically decreased the DA levels in the striatum, but not those in the other regions in normal healthy mice, and TNF-α and IL-1β levels were elevated in the striatum of MPTP/CLP-treated mice in comparison to Vcl/CLP-treated mice. SKF overcame the deteriorating effects of MPTP on the survival of septic mice, suggesting that SKF compensates for the reduction in striatal DA levels. Fenoldopam, a D1 selective agonist^27^^,^^28^ that does not cross the BBB, did not ameliorate mortality in septic mice. These results also suggest that SKF activity in the brain, rather than in the peripheral organs, is critical for the amelioration of sepsis.

Sepsis is typically accompanied by disturbance of the HPA axis,^38^ which is likely a cause of sleep disturbances.^39^ TNF-α and IL-1β are known to increase sleep duration,^40^^,^^41^ but they also have been shown to stimulate CRH and AVP synthesis and release in HPT.^42^^,^^43^^,^^44^ CRH increases the EEG frequency, leading to wakefulness or insomnia in rodents^45^^,^^46^ and humans.^47^ AVP has also been implicated in sleep disturbances or insomnia.^48^ Therefore, the effects of cytokines on sleep are controversial. In this study, SKF-treated septic mice displayed a prolonged sleeping period in comparison to Vcl/CLP mice, suggesting that the prevention of the expression of CRH and AVP by SKF strengthens the sleep-promoting effects of TNF-α and IL-1β.

The mechanisms by which neuroinflammation suppresses peripheral systemic inflammation remain to be elucidated. It may be possible that CLP and/or SKF-induced changes in the HPA axis were the cause of aggravated or suppressed inflammation in the peripheral tissues. A CLP-induced increase in the expression of CRH and AVP leads to the increased expression of POMC in the pituitary.^49^ The administration of SKF abolished this increase in the expression of POMC. However, the ACTH levels in the circulation of Vcl/CLP mice were lower than those in SKF/CLP mice. Although POMC should be cleaved into ACTH by prohormone convertase I, its expression is suppressed in septic mice.^34^ Furthermore, sepsis increases the expression of annexin A_I_ in the pituitary, which mediates the negative feedback effect of glucocorticoids. Thus, despite the higher expression of CRH/AVP and POMC, the circulating ACTH levels in Vcl/CLP mice were lower than those in CNT and SKF/CLP mice. The reason why the corticosterone levels in the circulation were almost the same between the Vcl/CLP and SKF/CLP mice, despite the different levels of ACTH, remains unclear. Corticosteroid binding globulin levels are reduced in proportion to the severity of sepsis, and therefore, glucocorticoid actions may have varied between Vcl/CLP and SKF/CLP mice despite the fact that the total corticosterone levels were almost the same.^50^ Collectively, the CLP-induced elevated expression of TNF-α and IL-1β leads to disturbances in the HPA axis at different levels, and SKF is considered to at least partially normalize the disturbances by reducing the expression of cytokines. CRH activates the sympathetic nervous system;^51^ however, there were no significant changes in adrenaline levels in the circulation of the Vcl/CLP and SKF/CLP mice.

Although the present data suggest that the anti-inflammatory effects of SKF may be mainly mediated through D1-like receptors in the brain, they may also be at least partly attributed to peripheral effects because D1R is widely expressed by several types of leukocytes,^52^ in which D1R activation may inhibit NLRP3 inflammasomes.^53^ The expression of D1R in human and murine polymorphonuclear cells has been reported,^54^ and a D1-like agonist has been shown to inhibit cell migration and ROS production. Furthermore, it is argued that DA is generated in the peripheral organs, including the lungs, affecting the immunological responses in these organs.^55^ These reports suggest that the anti-inflammatory effects in the peripheral tissues of SKF may arise independently of the effects in the brain.

The microglia may play a crucial role in CLP-induced neuroinflammation responsible for SAE.^22^^,^^56^ However, blood-borne macrophages invading the brain during sepsis pathology may be more aggravating than activated microglial cells, as shown in our previous study using a traumatic brain injury model.^12^^,^^14^ Astrocytes may also be involved in the neuroinflammation with significant expression of TNF-α and IL-1β.^17^ Microglia sorted from the brains of septic mice showed the elevated expression of TNF-α but not IL-1β, suggesting the possible involvement of the proinflammatory actions of macrophages and/or astrocytes.

Elevation of intracellular cAMP levels has been implicated in anti-inflammatory effects on microglia, and ligands for β2 adrenergic receptors have anti-inflammatory effects.^19^^,^^20^ Similarly, SKF binds to D1-like receptors, leading to elevation of intracellular cAMP, which may be mechanistically related to the anti-inflammatory actions of SKF.^22^ Although NA is another candidate for preventing neuroinflammation by elevating intracellular cAMP levels in microglia and macrophages, mainly through the β2 adrenergic receptor,^36^ there were no significant changes in the levels of NA in the present CLP-induced sepsis mouse model, denying the possibility that NA is involved in the suppression of SAE pathological processes.

Delirium is a typical symptom of cognitive dysfunction and a common consequence of SAE, occurring in approximately 50% of patients.^57^ SAE often becomes chronic and develops into dementia symptoms in sepsis survivors.^13^^,^^58^^,^^59^ TNF-α and IL-1β are thought to be critically involved in the pathogenesis of SAE. SKF ameliorated cognitive dysfunction in septic mice, as revealed by MWM and Y-maze tests. This favorable effect of SKF may also be attributed to its suppressive effects on TNF-α and IL-1β expression. The main pathology of Parkinson’s disease is the progressive degeneration of DArgic neurons in the substantia nigra, causing a severe decrease in DA levels in the striatum. It has been reported that patients were more prone to sepsis than controls,^60^ although whether the tendency to develop sepsis is related to the decrease in DA levels in the brain is unclear.

In conclusion, the favorable effects of the D1-like receptor selective agonist SKF on septic mice may be attributed to the suppression of neuroinflammation, but not inflammation in the peripheral tissues, due to the high expression of D1R and D5R in the brain. SKF ameliorated ALI-like pathology in CLP-induced septic mice, although the lungs did not significantly express D1-like receptors compared to the brain. The BBB-impermeable D1R agonist fenoldopam was ineffective at preventing CLP-induced death in animals. MPTP, a DArgic neuron toxicant in the brain, worsens mortality in septic animals. SKF abolished the CLP-induced elevated expression of CRH and AVP with increasing sleep duration and ameliorated cognitive dysfunction. These results suggest that the neuroinflammation that causes SAE may be the central mechanism underlying sepsis-related death. Clinically available D1-like receptor-selective agonists may be promising medicines for preventing sepsis-related death.

### Limitations of the study

Although the results suggest that SKF ameliorated sepsis by suppressing neuroinflammation, the effects of SKF on peripheral tissues remain to be elucidated. The mechanisms underlying SKF-mediated amelioration of ALI have not yet been clarified. MPTP treatment caused a significant decrease in DA levels only in the striatum. It is yet to be determined whether the suppression of the immunoreaction in the striatum by SKF where abundantly expressed D1R was the central cause of the improved survival of septic mice. Furthermore, only 7- to 8-week-old C57BL/6J male mice were used in this study, thereby, it was not investigated how sex, age, and animal strains affected the results.

## STAR★Methods

### Key resources table

**Table:** 

REAGENT or RESOURCE	RESOURCE	IDENTIFIER
**Experimental models: Organisms/strains**		

C57BL/6	CLEA Japan, Inc.	C57BL/6JJcl

**Chemicals, peptides, and recombinant proteins**		

SKF-81297 hydrobromide	Tocris Bioscience	Catalog number: 1447/10
SCH-23390 hydrochloride	Tocris Bioscience	Catalog number: 0925/10
Dimethyl Sulfoxide	FUJIFILM-Wako	Product number: 046-21981
Fenoldopam	Selleck Chemicals	Product code: S2089
Dopamine Hydrochloride	LKT Laboratories	Item number: LKT-D5662
MPTP	FUJIFILM-Wako	Product number: 136-16381
Normal saline	Otsuka	Product number: K3D88
Soldem 3A Infusion	Terumo	Product number: 230330KA
Phosphate Buffered Saline	FUJIFILM-Wako	Product number: 162-19321
0.2 mol/l Hydrochloric Acid	FUJIFILM-Wako	Product number: 080-02725
0.5M EDTA	Nippon Gene	Product number: 311-90075
Noradrenaline bitartrate	Tocris Bioscience	Product number: 5169/50
Serotonin	Toronto Research Chemicals	Product number: H977043
Paraformaldehyde Phosphate	FUJIFILM Wako	Product number: 161-20141
FBS	Nichirei Biosciences	Product number: 174012
BD FACS™ Lysing solution	BD Biosciences	Product number: 349202
CellCover	Anacyte	Reference Number: 800-250
Hoechst 33342	Thermo Fisher Scientific	Product Number: H1399

**Oligonucleotides**		

*Avp*	Hokkaido System Science CO	Accession Number: NM_009732.2
*Actb*	Hokkaido System Science CO	Accession Number: NM_007393.5
*Ccl2*	Hokkaido System Science CO	Accession Number: NM_011333.3
*Cd86*	Hokkaido System Science CO	Accession Number: NM_019388.3
*Crh*	Hokkaido System Science CO	Accession Number: NM_205769.3
*Crhr1*	Hokkaido System Science CO	Accession Number: NM_007762.5
*Crhr2*	Hokkaido System Science CO	Accession Number: NM_001288618.1
*Drd1*	Hokkaido System Science CO	Accession Number: NM_010076.3
*Drd2*	Hokkaido System Science CO	Accession Number: NM_010077.3
*Drd3*	Hokkaido System Science CO	Accession Number: NM_007877.2
*Drd4*	Hokkaido System Science CO	Accession Number: NM_007878.4
*Drd5*	Hokkaido System Science CO	Accession Number: NM_013503.4
*Gapdh*	Hokkaido System Science CO	Accession Number: NM_008084.4
*Il1b*	Hokkaido System Science CO	Accession Number: NM_008361.4
*Il6*	Hokkaido System Science CO	Accession Number: NM_031168.2
*Pomc*	Hokkaido System Science CO	Accession Number: NM_001278581.1
*Tnf*	Hokkaido System Science CO	Accession Number: NM_013693.3

**Critical commercial assays**		

Hematoxylin and Eosin Stain Kit	Vector laboratories	Product number:: H-3502
Mouse IL-1 beta ELISA Kit	Proteintech	Catalog number: KE10003
Mouse TNF-alpha ELISA Kit	Proteintech	Catalog number: KE10002
Mouse MCP-1 ELISA Kit	Proteintech	Catalog number: KE10006
Pierce BCA Protein Assay Kit	Thermo Fisher Scientific	Catalog number: 23225
Mouse ACTH ELISA Kit	Elabscience	Catalog number: E-EL-M0079
Mouse CORT ELISA Kit	Elabscience	Catalog number: E-EL-0161
Mouse EPI ELISA Kit	Elabscience	Catalog number: E-EL-0045
Zombie Violet™ Fixable Viability Kit	BioLegend	Catalog number: 423114
Adult Brain Dissociation Kit	Miltenyi Biotec	Catalog number: 130-107-677
Lung Dissociation Kit	Miltenyi Biotec	Catalog number: 130-095-927
Maxwell® RSC simplyRNA Cells / Tissue Kit	Promega	Catalog number: AS1390
RNeasy micro kit	Qiagen	Catalog number: 74004
qPCR RT Master Mix with gDNA Remover	Toyobo	Code number: FSQ-301
THUNDERBIRD® Next SYBR™ qPCR Mix	Toyobo	Code number: QPX-201

**Antibodies**		

APC/Cyanine7 anti-mouse CD3 Antibody	BioLegend	Catalog number: 100222; RRID: AB_2242784
APC anti-mouse/human CD11b Antibody	BioLegend	Catalog number: 101212; RRID: AB_312795
Pacific Blue™ anti-mouse/human CD11b Antibody	BioLegend	Catalog number: 101224; RRID: AB_755986
APC anti-mouse CD11c Antibody	BioLegend	Catalog number: 117310; RRID: AB_313779
APC anti-mouse CD19 Antibody	BioLegend	Catalog number: 115512; RRID: AB_313647
PE anti-mouse CD45 Antibody	BioLegend	Catalog number: 103106; RRID: AB_312971
Pacific Blue™ anti-mouse CD45 Antibody	BioLegend	Catalog number: 103126; RRID: AB_493535
APC/Cyanine7 anti-mouse NK-1.1 Antibody	BioLegend	Catalog number: 108724; RRID: AB_830871
APC anti-mouse Ly-6C Antibody	BioLegend	Catalog number: 128016; RRID: AB_1732076
PerCP anti-mouse Ly-6G Antibody	BioLegend	Catalog number: 127654; RRID: AB_2616999
Mouse anti-dopamine D1R/DRD1 Antibody	Novus Biologicals	Catalog number: NB110-60017; RRID: AB_905382
Rabbit anti-Iba1 Antibody	FUJIFILM-Wako	Product number: 012-28521; RRID: AB_2936184
Guinea Pig anti-NeuN Antibody	Merck	Catalog number: ABN90; RRID: AB_1120559
Alexa Fluor 488 donkey anti-mouse	Jackson ImmunoResearch Laboratories	Code: 715-546-020; RRID: AB_2340848
Cy3 donkey anti-rabbit	Jackson ImmunoResearch Laboratories	Code: 711-166-152; RRID: AB_2313568
Cy5 Donkey Anti-Guinea Pig	Jackson ImmunoResearch Laboratories	Code: 706-175-148; RRID: AB_2340462
Rat anti-mouse CD16/CD32 antibody	BD Biosciences	Catalog number: 553142; RRID: AB_394656

**Software and algorithms**		

Analysis Application Hybrid cell count	Keyence	BZ-H4C
FlowJo software program	FlowJo LLC	Version 7.6.5
Microscopy Image Analysis Software	Oxford Instruments	Imaris
Ethovision XT	Noldus Information Technology	XT 14
Sleep Sign	Kissei Comtec	Version 3
Prism software program	GraphPad Software	Version 8

### Resource availability

#### Lead contact

Further information and requests for resources and reagents should be directed to and will be fulfilled by the lead contact, Junya Tanaka, Email: jtanaka@m.ehime-u.ac.jp.

#### Materials availability

All resources and reagents used in this study is commercially available. This study did not generate new unique reagents.

#### Data and code availability

• All data in this study are available and found in Table S1 as an Excel file. The data has also been deposited in Mendeley Data, https://doi.org/10.17632/m95nhmctk8.1.
• This paper does not report original code.
• Any additional information will be fulfilled with the lead contact.

### Experimental model and study participant details

Murine sepsis model: Murine sepsis model was prepared using seven- to eight-week-old male C57BL/6J mice purchased from CLEA Japan Inc. (Shizuoka, Japan) by CLP technique. This study does not employ any higher vertebrate model or human study participants.

### Method details

#### Animals

Seven- to eight-week-old C57BL/6J male mice were purchased from CLEA Japan, Inc. (Shizuoka, Japan). Mice were acclimatized to the facility with 4-6 animals per cage under a 12-h light/dark cycle (lights on: 07:00, lights off: 19:00) at room temperature (25 ± 1°C). Food and water were provided *ad libitum*. All procedures were performed in accordance with the guidelines of the Ehime University Ethics Committee for Animal Experiments, Matsuyama, Japan and were approved by the Ethics Committee for Animal Experiments (approval number: 05U49-1, 05HO4-1).

#### CLP-induced sepsis model

The murine sepsis model was prepared using the CLP procedure.^30^ Mice were anesthetized using 3% isoflurane inhalation. The lower quadrants of the abdomen were soaked with a disinfectant, a longitudinal midline incision was made using scissors to extend the incision into the peritoneal cavity, and the cecum was located and exteriorized. The cecum was tightly ligated, penetrated two locations in the cecum using a 23-gauge needle, and gently squeezed to excrete a small amount of fecal material. The cecum was returned to the peritoneal cavity and the skin was sutured. The survival of the operated mice was monitored every 12 h for two weeks. Body temperature was measured over time using an environment logger (AD-1687; A&D Company, Tokyo, Japan) and an AX-KO4746-100 (A&D Company). In some experiments using MPTP, mice subjected to CLP were warmed on a heat pad for 1 h to suppress CLP-induced death.^30^ The viability of the CLP-treated mice was investigated for up to 22 days.

#### Pharmacological interventions

The D1-like receptor selective agonist SKF-81297 hydrobromide (SKF) and the D1-like receptor selective antagonist SCH-23390 hydrochloride (SCH; Tocris Bioscience, Bristol, UK) were dissolved in DMSO and stored at -30°C until use. Both SKF and SCH were diluted with 250 μg/ml 2.5% DMSO in saline. Saline containing 2.5 % DMSO was used as a vehicle. Both SKF and SCH were intraperitoneally administered to septic mice at a dose of 1.0 mg/kg body weight shortly after CLP and subsequently administered once/day (SKF) or twice/day (SCH) for up to a week. The vehicle was administered to CLP mice for up to 1 week as a control experiment. Fenoldopam (Selleck Chemicals, Tokyo, Japan), dissolved in the same way as SKF, was intraperitoneally administered at a dose of 10 mg/kg body weight with the same administration schedule as SKF. In some experiments, the animals were sacrificed at 6, 12, or 24 h after a single administration of SKF or fenoldopam. Doses were determined according to precvious literature.^22^^,^^61^^,^^62^

#### MPTP treatment

MPTP (FUJIFILM Wako, Osaka, Japan) was purchased and stored at room temperature. It was dissolved in saline (6.25 mg/ml) and injected subcutaneously into mice at 25 mg/kg body weight once per day for 5 days.^26^ The animals were kept in cages for seven days for recovery and subsequently subjected to CLP and pharmacological treatment with SKF.

#### Dissection of brain tissues

Dissected mouse brains were coronally sliced (thickness: 2 mm) using a brain slicer, and the frontal cortex (FC), dorsal (DStr) and ventral striatum (VStr), hippocampus (Hip), hypothalamus (HPT), and ventral midbrain (VMB) containing the substantia nigra and ventral tegmental area were obtained for qPCR and monoamine measurements. For some experiments, 1-mm-thick slices of the prefrontal cortex were prepared.

#### Complete blood count (CBC)

Following 100% carbon dioxide induced euthanasia, the blood samples obtained by cardiac puncture were subjected to a CBC using a Celltac Alpha MEK-6450 hematology analyzer (Nihon Kohden, Tokyo, Japan).^30^

#### Histological analyses with hematoxylin and eosin

The lungs were dissected and fixed by soaking the tissues in phosphate buffered saline (PBS) containing 4% paraformaldehyde. The fixed lung tissues were embedded in paraffin for sectioning (5 μm-thick) and stained with hematoxylin and eosin (HE). The specimens were observed and analyzed using a microscope BZ-X800 and a hybrid cell count software (Keyence, Osaka, Japan).

#### An enzyme-linked immunosorbent assay (ELISA)

Plasma samples were obtained from peripheral blood collected by centrifuging at 1000 *g* for 15 min at 4°C. TNF-α, IL-1β, and CCL2 levels in the plasma samples were determined using ELISA kits (Proteintech, Rosemont, IL, USA). Similarly, cytokine content in the hippocampal tissue was quantified. For brain tissue, an additional step was taken where the protein content of brain tissues was quantified using a Pierce BCA Protein Assay Kit (Thermo Fisher Scientific, Waltham, MA, USA). Plasma ACTH, corticosterone, and adrenaline levels were determined using an enzyme-linked immunosorbent assay (ELISA) kit (Elabscience, Houston, TX, USA).

#### High-performance liquid chromatography (HPLC)

Noradrenaline (NA), serotonin (5HT), and DA contents in the right frontal cortex were determined using HPLC.^63^ Tissues were homogenized in 0.2 M perchloric acid containing 5 mM ethylenediaminetetraacetic acid and 3,4-dihydroxybenzamine (FUJIFILM-Wako) with an ultrasonic cell disruption device. The homogenized tissue was filtered and aliquots were injected into an HPLC system with a reversed-phase column (Shimadzu Corporation, Kyoto, Japan). Synthetic NA, 5HT, and DA (FUJIFILM-Wako) were used as the standards. The monoamine content was normalized to the tissue protein content as measured using the Pierce BCA Protein Assay Kit.

#### Flow cytometry (FCM) and fluorescence-activated cell sorting (FACS)

FCM analyses and cell sorting for blood^30^ and tissue^64^ cells were performed as previously described. lood samples were mixed with PBS, and red blood cells were lysed using the BD Pharm Lyse solution (BD Biosciences, Franklin Lakes, NJ, USA). Zombie Violet (BD Biosciences) treatment was performed to eliminate dead cells. The samples were then incubated on ice with an anti-mouse CD16/CD32 antibody (BD Biosciences, 1:100) to block Fc receptors. The cells were incubated with the fluorescence-labelled antibodies listed in key resources table on ice for 30 min. After centrifugation, the pellets were resuspended in 250 μl of PBS containing 2% fetal bovine serum. Flow cytometry was performed using a Gallios flow cytometer (Beckman Coulter). The data were analyzed using FlowJo software (version 7.6.5; Treestar, Ashland, OR, USA). For FCM analysis of brain and lung tissues, tissues were dissociated into single cells using their respective dissociation kit following the manufacturer’s instructions (Miltenyi Biotec, Bergisch Gladbach, Germany), and the dissociated cells were processed as blood samples using the same method. Total RNA was collected from sorted cells and fixed overnight with a cell cover (Anacyte Laboratories, Hamburg, Germany).^14^

#### Quantitative reverse transcription polymerase chain reaction (RT-PCR; qPCR)

Total RNA was prepared from tissues using the Maxwell RSC simplyRNA Tissue Kit (Promega, Madison, WI, USA) and sorted using an RNeasy Micro Kit (Qiagen, Hilden, Germany). cDNA was synthesized using ReverTra Ace qPCR RT Master Mix with gDNA Remover (Toyobo, Osaka, Japan). qPCR was performed using the THUNDERBIRD Next SYBR qPCR Mix (Toyobo) with an MJ mini instrument (Bio-Rad, Hercules, CA, USA).^65^ All gene-specific mRNA expression levels were normalized to glyceraldehyde 3-phosphate dehydrogenase (GAPDH) and β-actin mRNA levels. All PCR primer sequences are listed in key resources table. All primers used in these studies showed a single peak on their melt curve. We also used positive and negative controls for each primer, where the negative controls did not show any signal. In most cases, two technical replicates and five to seven biological replicates were used, where differences between technical replicates >0.5 were considered to repeat qPCR. Ct value more than 36 for any gene were not considered for further analysis.

#### Immunofluorescence histochemistry

The primary antibodies listed in key resources table were used for histochemical staining.^66^ Mice were fixed by perfusion with 4% paraformaldehyde solution, and the fixed brains were cryo-sectioned into 10-mm-thick coronal sections at the frontal cortex level. After incubation with primary antibodies, the sections were incubated with DyLight 488, DyLight 549, and/or DyLight 649-labeled secondary antibodies (Jackson ImmunoResearch Laboratories, West Grove, PA, USA). Hoechst 33342 (Sigma-Aldrich) was used for nuclear staining. Imaging was performed using a TiE-A1R confocal microscope (Nikon, Tokyo, Japan) with a 100× objective lens (Plan Apo, NA: 1.45, Nikon). Images were analyzed using IMARIS ver. 9.8.2 (Oxford Instruments, UK).^67^

#### Behavioral tests

Behavioral tests were performed according to the schedule shown in Figure 7. The cognitive function of the mice was evaluated using the Morris water maze (MWM) and Y-maze tests. In the Y-maze test, the three arms of the test apparatus were labeled A, B, and C. A mouse was placed at the end of one arm, with the nose facing the center, and was allowed to explore for 5 min. The starting arms were assigned to animals in a pseudo-random manner; therefore, an equal number of mice from each group started from each arm. Mouse movements were recorded using a video camera located above the center of the maze and were analyzed using a video tracking system (Ethovision XT 14; Noldus Info. Tech., Wageningen, Netherlands). The order of arm entries was recorded, and triplets of the respective alterations were counted: correct alternations (e.g., ABC, BCA, CBA) and incorrect alternations (e.g., ABB, BBC, CCA). The percentage of correct alternations in the total number of arm entries was used as an index of spatial working memory.^68^ Mobile activities were evaluated with an open field test (OFT) using a 60-cm square arena with 40-cm high walls.^64^ The MWM test was performed using a round pool (1.2 m in diameter and 0.4 m in height) with water at 25 cm depth. Mice were trained three times a day at 20-min intervals for 2 days. In each trial, the mice were given 120 seconds to find a translucent acrylic platform with a diameter of 6 cm. On the third day, a probe test without the target platform was performed and the duration and frequency in the nearby zone, which is the quadrant of the pool where the platform was placed, were measured. In the probe test, mice were released into the water from the quadrant opposite to the location where the platform had been set. The mice swam freely for 120s, and the number of passes and the duration spent in the target quadrant were recorded.

#### Electroencephalogram (EEG)/electromyogram (EMG) evaluations

EEG/EMG was performed.^64^ Head mounts for EEG recordings (Pinnacle Technology, Inc., New York, NY, USA) were placed in the skull of mice (8 weeks old). Platinum-iridium double electrodes attached to the head mount were inserted bilaterally into the neck and back to monitor EMG activity. After surgery, the mice were allowed to fully recover over a three-week period prior to recording. EEG/EMGs were analyzed, and the percentage of wakefulness, non-rapid eye movement (NREM), and REM sleep durations were analyzed using the software program Sleep Sign ver. 3 (Kissei Comtec).

### Quantification and statistical analysis

Data are expressed as mean ± standard deviation. For statistical analyses, one of the following methods was employed: a two-tailed unpaired *Student’s or Welch’s* t-test, a one- or two-way analysis of variance (ANOVA) with Tukey’s or Sidak’s multiple comparisons test, or the Kaplan-Meier method with a log-rank test (Mantel-Cox) using Prism software (GraphPad Software, La Jolla, CA, USA). The statistical methods employed are described in the figure legends, P-values <0.05 were considered statistically significant. All the relevant statistical information is shown in Table S1.

## Supplemental information

Document S1. Figures S1 and S2

Table S1. All data presented and statistical analyses and information, related to all Figures
