# Supplementary material for: A dopamine D1-like receptor-specific agonist improves the survival of septic mice
Source: iScience. 2024 Mar 27;27(4):109587. doi: 10.1016/j.isci.2024.109587 (PMC11016908; doi:10.1016/j.isci.2024.109587)
Supplement: Document S1. Figures S1 and S2 [file mmc1.pdf]

iScience, Volume *27*

Supplemental information

A dopamine D1-like receptor-specific agonist improves the survival of septic mice

Koichi Tanaka, Mohammed E. Choudhury, Satoshi Kikuchi, Ikuko Takeda, Kensuke Umakoshi, Noriyuki Miyaue, Kanta Mikami, Ayane Takenaga, Harumichi Yagi, Rintaro Shinabe, Hironori Matsumoto, Hajime Yano, Masahiro Nagai, Jun Takeba, and Junya Tanaka

Supplemental Figures

Figure S1

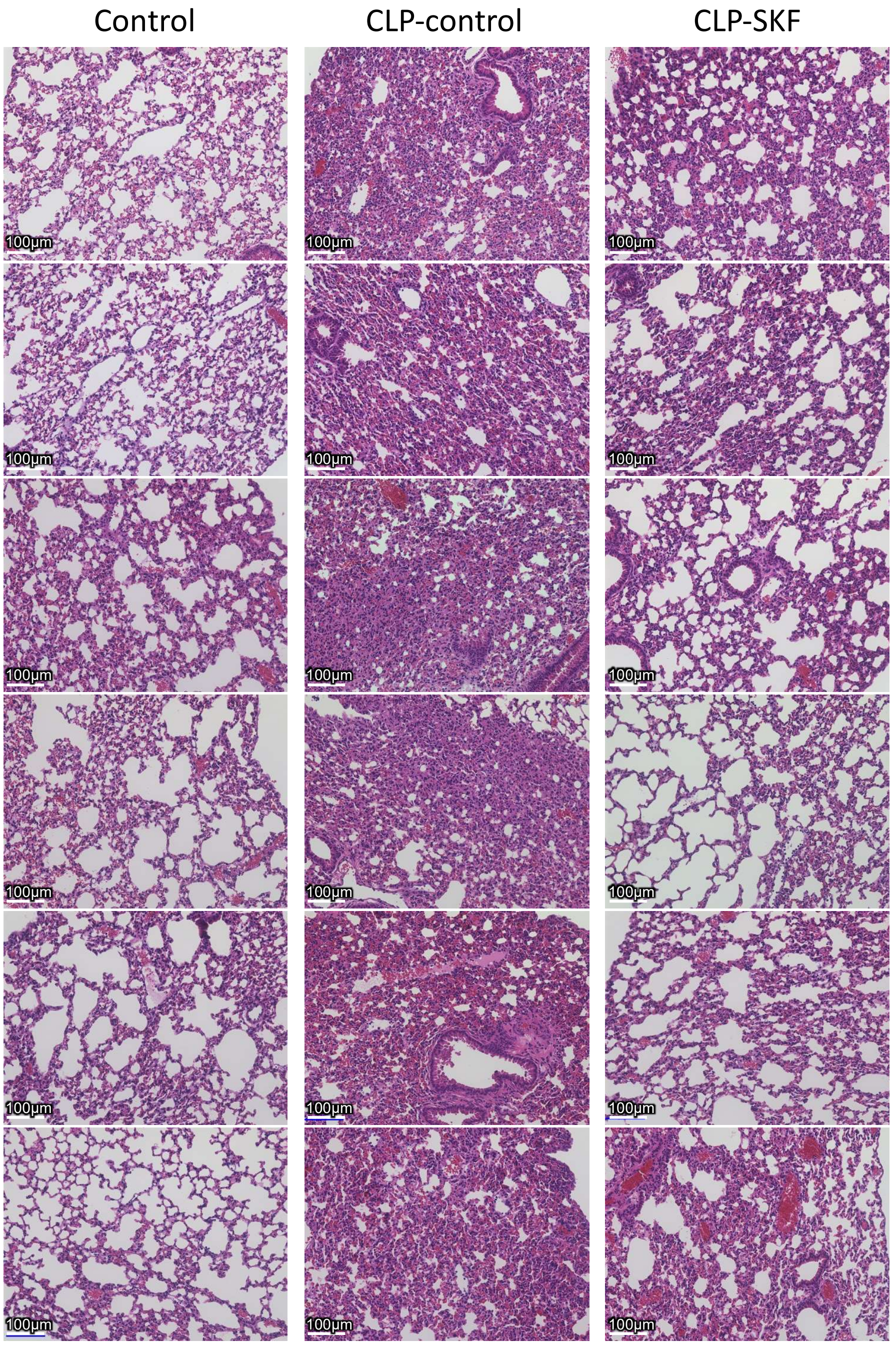

Figure S1: The lungs were dissected 24 h after CLP and subjected to fixation, slicing, and HE staining related to Figure 5, panel C.

Figure S2

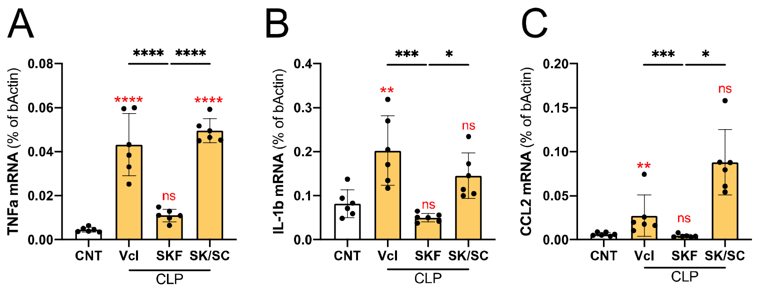

Figure S2: There are no significant differences between the data normalized with GAPDH-mRNA and those with β-actin-mRNA, related to Figure 6, panel G.
